# Development and Validation of Machine Learning Models in Prediction of Remission in Patients With Moderate to Severe Crohn Disease

**DOI:** 10.1001/jamanetworkopen.2019.3721

**Published:** 2019-05-10

**Authors:** Akbar K. Waljee, Beth I. Wallace, Shirley Cohen-Mekelburg, Yumu Liu, Boang Liu, Kay Sauder, Ryan W. Stidham, Ji Zhu, Peter D. R. Higgins

**Affiliations:** 1 Michigan Integrated Center for Health Analytics and Medical Prediction, Ann Arbor; 2 VA Center for Clinical Management Research, VA Ann Arbor Health Care System, Ann Arbor, Michigan; 3 Division of Gastroenterology and Hepatology, Department of Internal Medicine, Michigan Medicine, Ann Arbor; 4 Division of Rheumatology, Department of Internal Medicine, Michigan Medicine, Ann Arbor; 5 Department of Statistics, University of Michigan, Ann Arbor

## Abstract

**Importance:**

Biological therapies have revolutionized inflammatory bowel disease management, but many patients do not respond to biological monotherapy. Identification of likely responders could reduce costs and delays in remission.

**Objective:**

To identify patients with Crohn disease likely to be durable responders to ustekinumab before committing to long-term treatment.

**Design, Setting, and Participants:**

This cohort study analyzed data from 3 phase 3 randomized clinical trials (UNITI-1, UNITI-2, and IM-UNITI) conducted from 2011 to 2015. Participants (n = 401) were individuals with active (C-reactive protein [CRP] measurement of ≥5 mg/L at enrollment) Crohn disease who received ustekinumab therapy. Data analysis was performed from November 1, 2017, to June 1, 2018.

**Exposures:**

All included patients were exposed to 1 or more dose of ustekinumab for 8 weeks or more.

**Main Outcomes and Measures:**

Random forest methods were used in building 2 models for predicting Crohn disease remission, with a CRP level lower than 5 mg/dL as a proxy for biological remission, beyond week 42 of ustekinumab treatment. The first model used only baseline data, and the second used data through week 8.

**Results:**

In total, 401 participants, with a mean (SD) age of 36.3 (12.6) years and 170 male (42.4%), were included. The week-8 model had a mean area under the receiver operating characteristic curve (AUROC) of 0.78 (95% CI, 0.69-0.87). In the testing data set, 27 of 55 participants (49.1%) classified as likely to have treatment success achieved success with a CRP level lower than 5 mg/L after week 42, and 7 of 65 participants (10.8%) classified as likely to have treatment failure achieved this outcome. In the full cohort, 87 patients (21.7%) attained remission after week 42. A prediction model using the week-6 albumin to CRP ratio had an AUROC of 0.76 (95% CI, 0.71-0.82). Baseline ustekinumab serum levels did not improve the model’s prediction performance.

**Conclusions and Relevance:**

In patients with active Crohn disease, demographic and laboratory data before week 8 of treatment appeared to allow the prompt identification of likely nonresponders to ustekinumab without the need for costly drug-level monitoring.

## Key Points

**Question:**

Can laboratory data be used to predict durable response to ustekinumab among patients with Crohn disease before they commit to long-term treatment?

**Findings:**

In this cohort study of 401 participants with active Crohn disease, a prediction model using demographic and laboratory data available after 1 dose of ustekinumab reliably identified patients with active Crohn disease who were likely to respond to treatment beyond week 42. The week-6 albumin to C-reactive protein ratio seemed to be a pragmatic predictor of future remission, but early ustekinumab serum levels appeared to add little to the predictive value of this model.

**Meaning:**

Long-term ustekinumab response may be predicted using demographic and laboratory data before week 8 of treatment, without the need for costly drug-level monitoring.

## Teaser

This cohort study describes 2 models for predicting the long-term response to ustekinumab therapy among patients with active Crohn disease.

## Introduction

Inflammatory bowel disease (IBD) has been estimated to affect more than 1.6 million people in the United States on the basis of the adjusted prevalence rates in the 2010 US white population.^1,2^ Although the advent of expensive biological medications revolutionized the treatment of IBD,^3^ the increasing use of these agents has revealed substantial heterogeneity of treatment effect across the population with IBD and likely overuse of these medications. Anti–tumor necrosis factor drugs, still the standard therapy for moderate to severe IBD, did not induce remission in 20% to 30% of patients and lost effectiveness within a year in another 30% to 40% of patients.^3,4,5,6^ Novel biological agents emerging in recent years can help some patients who do not respond to anti–tumor necrosis factor therapy,^6^ but these agents are ineffective in many others.^7^ Initial efforts to improve remission rates with these agents have focused on optimizing serum drug levels and screening for antidrug antibodies, and these efforts have had some success.^3,5^

Interest has been growing in personalizing treatment strategies by using methods such as machine learning to match patients with IBD to the treatment most likely to work for them. These techniques using clinical and laboratory predictors that outperform drug levels alone may decrease avoidable drug costs, hospitalizations, surgical procedures, and complications while allowing patients with IBD to achieve symptomatic and biological remission much sooner than the typical 52-week clinical trial end point.^8,9^ Such approaches have not yet been applied to ustekinumab. Previous studies of ustekinumab pharmacokinetics present mixed findings. Some suggest a dose-related association between serum drug concentrations of ustekinumab and treatment efficacy in Crohn disease similar to that seen with other novel agents^8^; others suggest that serum drug concentrations of ustekinumab are relatively poor predictors of clinical response in Crohn disease.^10^

The purpose of the present study was to (1) identify characteristics of patients with Crohn disease at baseline and at week 8 of ustekinumab therapy (before the start of subcutaneous maintenance therapy) that predict long-term remission on ustekinumab therapy using C-reactive protein (CRP) level as a biomarker of disease activity, and (2) evaluate the incremental advantage of using these predictors compared with drug level alone.

## Methods

We used publicly available data from 3 randomized clinical trials enrolling patients with active Crohn disease, UNITI-1 (ClinicalTrials.gov identifier NCT01369329), UNITI-2 (identifier NCT01369342), and IM-UNITI (identifier NCT01369355), conducted from 2011 to 2015. Trials UNITI-1 and UNITI-2 were conducted at 178 sites in 23 countries and 175 sites in 23 countries respectively. The IM-UNITI trial was conducted at 260 sites in 27 countries.^7^ These data sets were obtained through the YODA (Yale Open Data Access) Project.^11^ The University of Michigan Institutional Review Board confirmed that institutional review board approval for this study was not necessary because it evaluated previously collected and deidentified phase 3 clinical trial data. Informed consent was not obtained for this reason. This study followed the Strengthening the Reporting of Observational Studies in Epidemiology (STROBE) reporting guideline and the Transparent Reporting of a Multivariable Prediction Model for Individual Prognosis or Diagnosis (TRIPOD) reporting guideline. Data analysis was performed from November 1, 2017, to June 1, 2018.

The original clinical trial cohort had 1409 participants, 668 of whom were either randomized to placebo or lost to follow-up by week 8 of the trial and therefore excluded. The remaining 741 participants received ustekinumab at week 8. Of these, 462 participants had CRP measurements of 5 mg/L or higher at enrollment, suggesting active disease, and 61 participants were excluded for missing values. The final cohort comprised 401 participants, whose data were used for the week-8 model and sensitivity analyses (eTable in the Supplement). The baseline model excluded 30 additional patients for missing baseline laboratory tests and included 371 patients (eFigure 1 in the Supplement).

### Predictor Variables

The week-8 model included 401 patients, 5 demographic predictors (age, sex, race, ethnicity, and weight), and laboratory tests performed at week 8 (CRP level at weeks 0, 3, 6, and 8; ratio of serum ustekinumab level to CRP level at week 0 after the first ustekinumab dose, week 3, and week 6 [eTable in the Supplement]). The week-8 drug level was not used in modeling because of missing values. We also tracked CRP measurement in the long term and calculated mean, maximum, minimum, and the slope of CRP level from weeks 0 to 3, weeks 3 to 6, and weeks 6 to 8.

The baseline model at week 0 included 371 patients, and 30 patients were excluded from this cohort owing to missing baseline laboratory tests. This model included the 5 demographic predictors and 10 laboratory predictors (CRP level; platelet count; hemoglobin level; absolute counts of leukocytes, monocytes, lymphocytes, eosinophils, segmented neutrophils, and basophils; and serum ustekinumab concentration measured approximately 1 hour after the first drug dose).

### Definition of Outcomes

The primary outcome was the most recent available CRP measurement taken after week 42, which we used as a surrogate biological end point for Crohn disease inflammatory activity in patients with baseline CRP elevation. We dichotomized the outcome into 2 categories: CRP level lower than 5 mg/L as evidence of Crohn disease remission and CRP level of 5 mg/L or higher as evidence of continued Crohn disease activity and thus treatment failure (to convert CRP to nanomoles per liter, multiply by 9.524).^12^ We chose this outcome because endoscopic data (the criterion standard for disease activity) were not available from the trial cohort of interest, and CRP level was the only objective disease activity measure available at multiple time points from the clinical trials.

No study visits or missing CRP measurements after week 42 were defined as treatment failures. We considered the utility of a subjective outcome measure, such as the Crohn Disease Activity Index (CDAI), but given the relatively low specificity of the CDAI, we instead chose an objective primary outcome variable. We also considered fecal calprotectin given its known high sensitivity over CRP for detection of active endoscopic disease activity.^13^ However, we were limited by missing fecal calprotectin data, which might bias our results; instead, we chose to include fecal calprotectin as an outcome in a sensitivity analysis.

### Statistical Analysis and Model Development

Random forest is a machine learning method of prediction that uses an ensemble of decision trees^14,15^ to classify observations. As previously described elsewhere,^8,9^ information from a single patient (an observation) is evaluated by many decision trees (a forest) built on different bootstrap samples of the original data set. Each tree classifies the observation independently, which can be viewed as that tree’s vote. The forest then combines the votes from the individual trees to generate a summary prediction. A cutoff value of the outcome variable is typically prespecified to provide the desired balance of sensitivity and specificity for the model. We used 500 trees to build each random forest model. Random forest modeling was chosen in this case because of its ability to model complex, nonlinear relationships and to easily accommodate complexities, such as interaction terms, which may not be captured by parametric models like logistic regression. This method has been used in previous studies.^8,9^

### Training and Testing Cohorts

As has been published,^8,9^ the baseline and 8-week data sets were each split randomly into a 70% training set for model development and a 30% testing set. This splitting process was replicated 100 times, and each time the model was fit on the training set and used to predict the outcome in the testing set. This 100-times replication process was used to generate an estimate of the variation of the area under the receiver operating characteristic curve (AUROC) computed on the testing set. The mean AUROC was then obtained from the 100 replication test sets. Subsequently, the 1 split that produced a representative AUROC that was closest to the mean AUROC for the week-8 model was selected as a representative split (training and testing cohorts) for both the week-8 and baseline models. This split was used to produce AUROC plots, cutoff point selection, and misclassification tables. These results were then compared across models.

The AUROC was used to evaluate the performance of each model. All statistical analyses were performed with the statistical language R, version 3.3, randomForest^14^ and pROC packages (R Foundation for Statistical Computing). The optimal cutoff was defined as the point on the receiver operating characteristic plot with the highest sensitivity and specificity (ie, the point at which [1 – sensitivity]^2^ + [1- specificity]^2^ is minimized).

### Variable Importance and Partial Dependence Plots

We assessed predictor variable importance using a random forest model trained on the entire data set at baseline and at week 8.^8,9^ We identified nodes in the group of trees in which each predictor appeared and then summed the information content of all nodes containing that predictor. Predictors providing greater combined discrimination were deemed to have higher importance. We built partial dependence plots on the entire cohort to demonstrate how each predictor affected the probability of success, defined by CRP level lower than 5 mg/L after week 42.^8,9^ For each predictor, we used the random forest model on the entire data set at that time point to predict the probability of success by patient, with that predictor set to a given value and averaged over all patients to produce a mean probability of success. We repeated this procedure for each value of that predictor in the data, or for its 50 quantiles if more than 50 values were available.

### Simpler Pragmatic Models and Sensitivity Analyses

Given the complexity of random forest models, simpler models with comparable performance are typically preferred for clinical use. Because CRP^16^ and albumin^17^ are biomarkers used in the management of IBD, we constructed pragmatic single-variable prediction models with both baseline and weeks 6 to 8 data. This decision could also be justified by these 2 predictors’ association with the variable importance plots. We evaluated these single-variable models on the full data set and compared the resulting predictions with those generated by the random forest models. We favored a simplified model that did not require week-8 data because a week-8 model would not allow clinicians to decide whether to continue ustekinumab until after the week-8 dose had been administered. To evaluate whether the outcome accurately represented Crohn disease activity, we performed a sensitivity analysis with the same variables used in the week-8 model to predict a dichotomized version of the patient’s last CDAI score after week 42, with 150 as the cutoff point. Missing values were viewed as treatment failures. We included week-6 fecal calprotectin as a covariate in the week-8 model to evaluate prediction performance for a CRP-based outcome. We also created a model substituting dichotomized fecal calprotectin after week 42 as the outcome of interest, with thresholds of 100 μg/g or 400 μg/g.

## Results

In total, 401 participants were included in the final analysis. Among the participants, the mean (SD) age was 36.3 (12.6) years and 170 (42.4%) were male. The original clinical trial cohort comprised 741 participants who received ustekinumab at week 8, whereas the present study’s week-8 model cohort had 401 participants and the baseline model cohort had 371. Additional demographic and clinical characteristics of all participants are presented in Table 1.

**Table 1.** Participant Demographics

Variable	Original Cohort (N = 741)	Modeling Cohort (N = 401)	Baseline Cohort (N = 371)
Age, mean (SD), y	37.7 (12.80)	36.3 (12.6)	36.3 (12.2)
Male sex, No. (%)	314 (42.)	170 (42.4)	158 (42.6)
White race, No. (%)	626 (84.5)	326 (81.3)	301 (81.1)
Weight, mean (SD), kg	71.0 (19.9)	70.2 (20.3)	70.3 (20.3)
Baseline CRP, median (IQR), mg/L	8.5 (3.4-22.2)	16.9 (9.2-32.3)	16.8 (9.3-32.8)
Concomitant medications, No. (%)			
Aminosalicylic acid	287 (38.7)	168 (41.9)	153 (41.2)
Immunosuppressants	308 (41.6)	170 (42.4)	152 (41.0)
Glucocorticoids	414 (55.9)	212 (52.9)	203 (54.7)
Location of disease, No. (%)			
Colon only	128 (17.3)	69 (17.2)	63 (17.0)
Ileum only	150 (20.2)	63 (15.7)	58 (15.6)
Colon and ileum	459 (61.9)	266 (66.3)	248 (66.8)
Perianal	273 (36.8)	176 (43.9)	167 (45.0)
Extraintestinal manifestation	387 (52.2)	214 (53.4)	203 (54.7)
Proximal small bowel	135 (18.2)	77 (19.2)	70 (18.9)

Abbreviations: CRP, C-reactive protein; IQR, interquartile range.

SI conversion factor: To convert CRP to nanomoles per liter, multiply by 9.524.

### Baseline Model and Predictors

**Figure 1.**
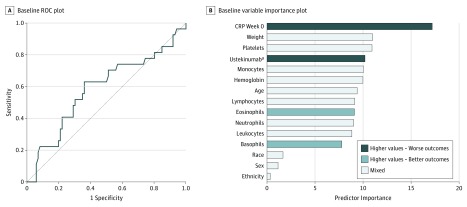
Baseline Predictors A, Receiver operating characteristic (ROC) plot for baseline model. The area under the ROC curve in the representative testing set was 0.59 (95% CI, 0.46-0.72). B, Baseline variable importance plot. The importance of each predictor was computed by identifying nodes in the trees and summing up all information provided by the nodes containing the predictor. The higher the importance, the larger the total discrimination provided by the predictor. CRP indicates C-reactive protein. ^a^Level 1 hour after first dose.

The mean (SD) AUROC for the baseline model in the testing set was 0.56 (0.053), and the AUROC in the representative testing set was 0.59 (95% CI, 0.46-0.72) (Figure 1A). The best predictors of CRP at week 0 in the baseline model were baseline CRP level, weight, platelet count, ustekinumab serum level after the first dose of drug, and monocyte count (Figure 1B). Race, sex, and ethnicity were weak predictors. Sensitivity for the baseline model, with 113 participants in the testing set, was 0.63, and specificity was 0.64. Table 2 lists the best cutoff values, number of predicted successes and failures, and prediction accuracy rates for the baseline model.

**Table 2.** Prediction Performances of Models

Model Tested	Test Set Size	AUROC (95% CI)	Optimal Cutoff	Predicted Cases		Accurate Prediction Rate		Sensitivity	Specificity	Brier Score^a^
				Success (≥Cutoff)	Failure (<Cutoff)	Success (≥Cutoff)	Failure (<Cutoff)			
Baseline model	113	0.59 (0.46-0.72)	0.24	48	65	0.35	0.85	0.63	0.64	0.18
Baseline CRP alone	401	0.67 (0.61-0.74)	14.65 (mg/L)	182	219	0.32	0.87	0.67	0.61	NA
Week-8 model	120	0.78 (0.69-0.87	0.26	55	65	0.49	0.89	0.79	0.67	0.17
Week-8 CRP alone	401	0.76 (0.71-0.82)	8.30	178	223	0.39	0.91	0.78	0.65	NA
Week-6 (albumin × ustekinumab-to-CRP ratio) model	398	0.71 (0.65-0.77)	24.71	177	221	0.33	0.88	0.69	0.62	NA
Week-6 CRP alone	401	0.75 (0.70-0.81)	6.77 (mg/L)	151	250	0.41	0.90	0.70	0.72	NA
Week-6 albumin to CRP ratio	398	0.76 (0.71-0.82)	4.92	166	232	0.40	0.91	0.77	0.68	NA

Abbreviations: AUROC, area under the receiver operating characteristic curve; CRP, C-reactive protein; NA, not applicable.

SI conversion factor: To convert CRP to nanomoles per liter, multiply by 9.524.

^a^ Brier score, with a range of 0 to 1, is a measure of calibration.

Partial dependence plots were created to illustrate the association between each predictor and the outcome (eFigure 2 in the Supplement). Most predictors showed nonlinear associations. The likelihood of treatment success (ie, CRP level lower than 5 mg/L after week 42) increased steeply with lower baseline CRP measurements, with baseline CRP measurements of 14.65 mg/L or lower predicting treatment success. Weight and platelet count did not show monotonic trends.

### Week-8 Model and Predictors

**Figure 2.**
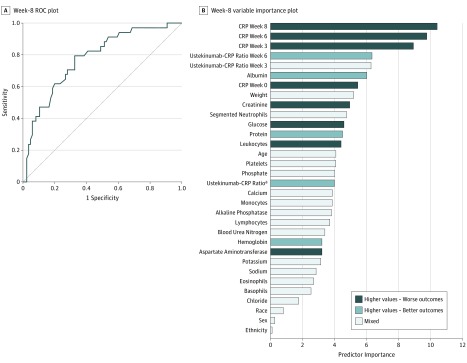
Week-8 Predictors A. Receiver operating characteristic (ROC) plot for week-8 model with C-reactive protein (CRP) and ustekinumab to CRP ratio. The area under the ROC curve in the representative testing set was 0.78 (95% CI, 0.69-0.87). B, Week-8 variable importance plot. The importance of each predictor was computed by identifying nodes in the trees and summing all information provided by the nodes containing the predictor. The higher the importance, the larger the total discrimination provided by the predictor. ^a^One hour after infusion drug level with closest prior CRP.

The mean (SD) AUROC for the week-8 model was 0.78 (0.042) (Figure 2A), and the AUROC on the representative testing set was 0.78 (95% CI, 0.69-0.87). The most important predictors in this model were CRP levels at weeks 3, 6, and 8 and serum ustekinumab to CRP ratio at weeks 3 and 6 (Figure 2B). Other than CRP, the most important laboratory variable included in this model was albumin measured at week 8. Race, sex, and ethnicity were weak predictors. Sensitivity for the week-8 model, with 120 participants in the testing set, was 0.79, and specificity was 0.67.

The week-8 model classified participants into 2 groups: those in whom treatment was predicted to succeed (n = 55 [45.8%]), and those in whom treatment was predicted to fail (n = 65 [54.2%]). Twenty-seven participants (49.1%) classified as likely to have treatment success achieved the goal of CRP level lower than 5 mg/L after week 42, and 7 of 65 participants (10.8%) classified as likely to have treatment failure achieved this outcome. For comparison, 87 participants (21.7%) of the full cohort achieved the specified outcome of remission after week 42.

### Predictors After a Single Dose of Ustekinumab

The probability of treatment success increased steeply with lower CRP levels at weeks 3, 6, and 8 (eFigure 3 in the Supplement). A higher value of the ustekinumab to CRP ratio at week 6 was associated with an increased treatment success rate, but the association was uncertain when the value was close to 0. The ustekinumab to CRP ratio at week 3 showed a nonmonotonic association with treatment success rate. A higher albumin level at week 6 was also associated with an increased treatment success rate. Other predictors showed weak or nonmonotonic associations with the outcome.

### Simpler Pragmatic Models

The predictive performance of the simpler pragmatic models evaluated on the full data set are shown in Table 2. We found that baseline CRP (prior to first ustekinumab dose) was a weak predictor of treatment success, with an AUROC of 0.67 (95% CI, 0.61-0.74). The week-6 albumin (grams per deciliter) to CRP (milligrams per liter) ratio was as accurate as week 8 CRP alone, with an AUROC of 0.76 (95% CI, 0.71-0.82) and a cutoff of 4.92. It was less accurate than the week-8 random forest model. A CRP value at week 6 or 8 also performed well, with an AUROC of 0.75 (95% CI, 0.70-0.81) at week 6 and 0.76 (95% CI, 0.71-0.82) at week 8. The CRP measurement at week 8 also predicted treatment success with reasonable accuracy in this cohort of patients with high baseline CRP level. However, it was inferior to the full model and to the week-6 albumin to CRP ratio by sensitivity, specificity, and accurate prediction rate. Adding the week-6 ustekinumab serum level to the week-6 albumin to CRP ratio worsened the accuracy of this pragmatic model. These models are compared in Table 2.

### Sensitivity Analysis for Clinical End Point

We fitted a random forest model with the same predictors used in the week-8 model, substituting the dichotomized CDAI score (<150 indicates clinical remission) after week 42 as the outcome of interest. Using this outcome definition, we identified 242 (60.3%) treatment failures and 159 (39.7%) treatment successes. Performance of the week-8 model was poor for this clinical end point, with an out-of-bag AUROC of 0.61 (95% CI, 0.55-0.66) for the CDAI end point.

Including week-6 fecal calprotectin value as a covariate in the week-8 model did not improve the prediction performance for a CRP-based outcome. The mean AUROC was 0.76 (95% CI, 0.71-0.82). Substituting the dichotomized fecal calprotectin value after week 42 as the outcome of interest with thresholds of 100 μg/g returned a mean (SD) AUROC of 0.64 (0.061) or 400 μg/g returned a mean (SD) AUROC of 0.63 (0.060).

## Discussion

Substantial heterogeneity in treatment response to costly biological therapies exists across the population with Crohn disease. For this reason, there is great interest in developing tools that can reliably identify patients who are unlikely to respond to costly medications after a short course of treatment. If effective, such tools could help clinicians identify patients who should be switched to an alternative treatment with a greater chance of success, reducing both the costs associated with an expensive but likely unsuccessful treatment and the harms associated with suboptimal disease control and avoidable steroid exposure.

We applied machine learning techniques to phase 3 trial data to generate predictive models of treatment response to ustekinumab at week 42 in patients with active Crohn disease, as defined by an elevated CRP level at enrollment using dichotomized CRP as a marker of biological remission. The model we constructed with baseline data was relatively inaccurate, performing worse than baseline CRP measurement alone (AUROC for CRP alone was 67%, with 67% sensitivity and 61% specificity). Thus, the baseline model should not be used clinically. This finding suggests, as do studies on vedolizumab,^8,9^ that predicting an individual’s response to a biological therapy before the first dose is difficult. The week-8 model provided the best performance characteristics for predicting long-term biological response with an AUROC of 0.78 (95% CI, 0.69-0.87). This model also proved able to distinguish long-term biological remission (as defined by CRP <5 mg/L beyond 42 weeks) from treatment failures. Simpler pragmatic models also showed good performance, with the week-6 albumin-to-CRP ratio showing an AUROC of 0.76 (95% CI, 0.71-0.82) at an albumin-to-CRP ratio cutoff of 4.92. Patients with higher values of this ratio at week 6 are increasingly likely to achieve biological remission.

Neither cross-sectional nor long-term measures of ustekinumab were associated with improvements in the performance of any of the models we tested, and they were associated with worse performance of week-6 pragmatic model. This finding supports previous studies demonstrating that serum ustekinumab level cannot adequately discriminate treatment responders from nonresponders in patients with Crohn disease enrolled in the UNITI clinical trials.^10^ It seems unlikely that increasing the frequency of ustekinumab dosing will provide additional advantages in early nonresponders.

Knowing this finding might help clinicians decide to switch drug classes or to augment ustekinumab with an anti–tumor necrosis factor or a Janus kinase inhibitor if a substantial objective improvement in inflammation is not seen before the second dose rather than commit additional time and resources to costly monotherapy intensification, which appears unlikely to be effective. This finding allows clinicians to limit futile care with expensive biological agents such as ustekinumab 90 mg, which has a list price of approximately $22 000 per dose.^18^ In clinical practice, either the week-8 random forest model or the pragmatic model using the week-6 albumin to CRP ratio could predict outcomes, although the pragmatic model appears more likely to be used at the point of care.

### Limitations

This study has several limitations. First, we caution that the prediction performances of the pragmatic models should not be directly compared with those of the full models given that the pragmatic models were developed and tested on the entire cohort, whereas the random forest models were developed on a 70% random sample of the cohort and tested on the remaining 30%. Prior to clinical use, the pragmatic models would, ideally, be externally validated using a separate cohort. Second, we used patients with Crohn disease selected for enrollment in a clinical trial, which might not be generalizable to all patients with Crohn disease. Third, CRP is not a perfect proxy for biological remission given that up to 20% of patients with Crohn disease do not experience elevated CRP levels during a flare. Endoscopic data, which were not available for this cohort, would be ideal. To reduce the implications of this limitation, we used only the data from patients with elevated CRP levels at baseline and in whom CRP is more likely to be a reliable biomarker. Although this decision improves the reliability of these results, it might limit generalizability to patients who received ustekinumab with an elevated baseline CRP levels. Fourth, before this model can be used in a real-world setting, long-term intervention studies are needed to evaluate the best course of action for patients deemed unlikely to achieve biological remission with ustekinumab. Fifth, this test, like any other diagnostic test, is imperfect, and clinical context should be considered when making decisions about its use and interpretation.

## Conclusions

In this study of clinical trial data, a model using demographic and laboratory data available after the first dose of ustekinumab reliably identified which patients with active Crohn disease at enrollment were likely to achieve treatment response beyond week 42. The week-6 albumin to CRP ratio is a potential predictor of future biological remission during ustekinumab therapy among patients with high baseline CRP levels. Unlike other biological agents for Crohn disease, ustekinumab serum did not appear to add substantial predictive value for future biological remission.

## Notes

Supplement. **eTable.** Quantitative Laboratory Tests Included in Week-8 Model **eFigure 1.** Consort Diagram **eFigure 2.** Partial Dependence Plots for Baseline Model Predictors **eFigure 3.** Partial Dependence Plots for Week-8 Model Predictors
