# Error in End Matter Disclaimer

**DOI:** 10.1001/jamanetworkopen.2019.7386

**Published:** 2019-06-28

In the Original Investigation titled “Development and Validation of Machine Learning Models in Prediction of Remission in Patients With Moderate to Severe Crohn Disease,”^1^ published May 10, 2019, there was an omission in the end matter Disclaimer. The Disclaimer should have ended with the following sentences: “In addition, this study, carried out under YODA Project 2016-1176, used data obtained from the Yale University Open Data Access Project, which has an agreement with Janssen Research & Development LLC. The interpretation and reporting of research using this data are solely the responsibility of the authors and do not necessarily represent the official views of the Yale University Open Data Access Project or Janssen Research & Development LLC.” This article has been corrected.^1^
