# Supplementary material for: Development and Validation of Machine Learning Models in Prediction of Remission in Patients With Moderate to Severe Crohn Disease
Source: JAMA Netw Open. 2019 May 10;2(5):e193721. doi: 10.1001/jamanetworkopen.2019.3721 (PMC6512283; doi:10.1001/jamanetworkopen.2019.3721)
Supplement: Supplement. — eTable. Quantitative Laboratory Tests Included in Week-8 Model eFigure 1. Consort Diagram eFigure 2. Partial Dependence Plots for Baseline Model Predictors eFigure 3. Partial Dependence Plots for Week-8 Model Predictors [file jamanetwopen-2-e193721-s001.pdf]

Supplementary Online Content

Waljee AK, Wallace BI, Cohen-Mekelburg S, et al. Development and validation of machine learning models in prediction of remission in patients with moderate to severe Crohn disease. *JAMA Netw Open.* 2019;2(5): e193721. doi:10.1001/jamanetworkopen.2019.3721

eTable. Quantitative Laboratory Tests Included in Week-8 Model

eFigure 1. Consort Diagram

eFigure 2. Partial Dependence Plots for Baseline Model Predictors

eFigure 3. Partial Dependence Plots for Week-8 Model Predictors

This supplementary material has been provided by the authors to give readers additional information about their work.

©2019 Waljee AK et al. *JAMA Network Open.*

eTable. Quantitative Laboratory Tests Included in Week-8 Model

Variable Name	Variable definition	Unit
CRP 8	C Reactive Protein at week 8	mg/L
CRP 6	C Reactive Protein at week 6	mg/L
CRP 3	C Reactive Protein at week 3	mg/L
Uste_6_Ratio_CRP	Uste drug level at week 6/ CRP at week 6	NA
Uste_3_Ratio_CRP	Uste drug level at week 3/ CRP at week 3	NA
CRP 0	C Reactive Protein at week 0 before first dose of treatment	mg/L
Albumin		g/L
Glucose		mmol/L
Neutrophils Segmented		10E9/L
Creatinine		umol/L
Protein		mmol/L
Leukocytes		10E9/L
Phosphate		mmol/L
Platelets		10E9/L
Uste_1_Ratio_CRP	Uste drug level at week 0 after first dose/ CRP at week 0 before first dose	NA
Monocytes		10E9/L
Calcium		mmol/L
Lymphocytes		10E9/L
Blood Urea Nitrogen		mmol/L
Eosinophils		10E9/L
Hemoglobin		g/L
Alkaline Phosphatase		U/L
Aspartate Aminotransferase		U/L
Potassium		mmol/L
Sodium		mmol/L
Basophils		10E9/L
Chloride		mmol/L

©2019 Waljee AK et al. *JAMA Network Open.*

eFigure 1. Consort Diagram

©2019 Waljee AK et al. *JAMA Network Open.*

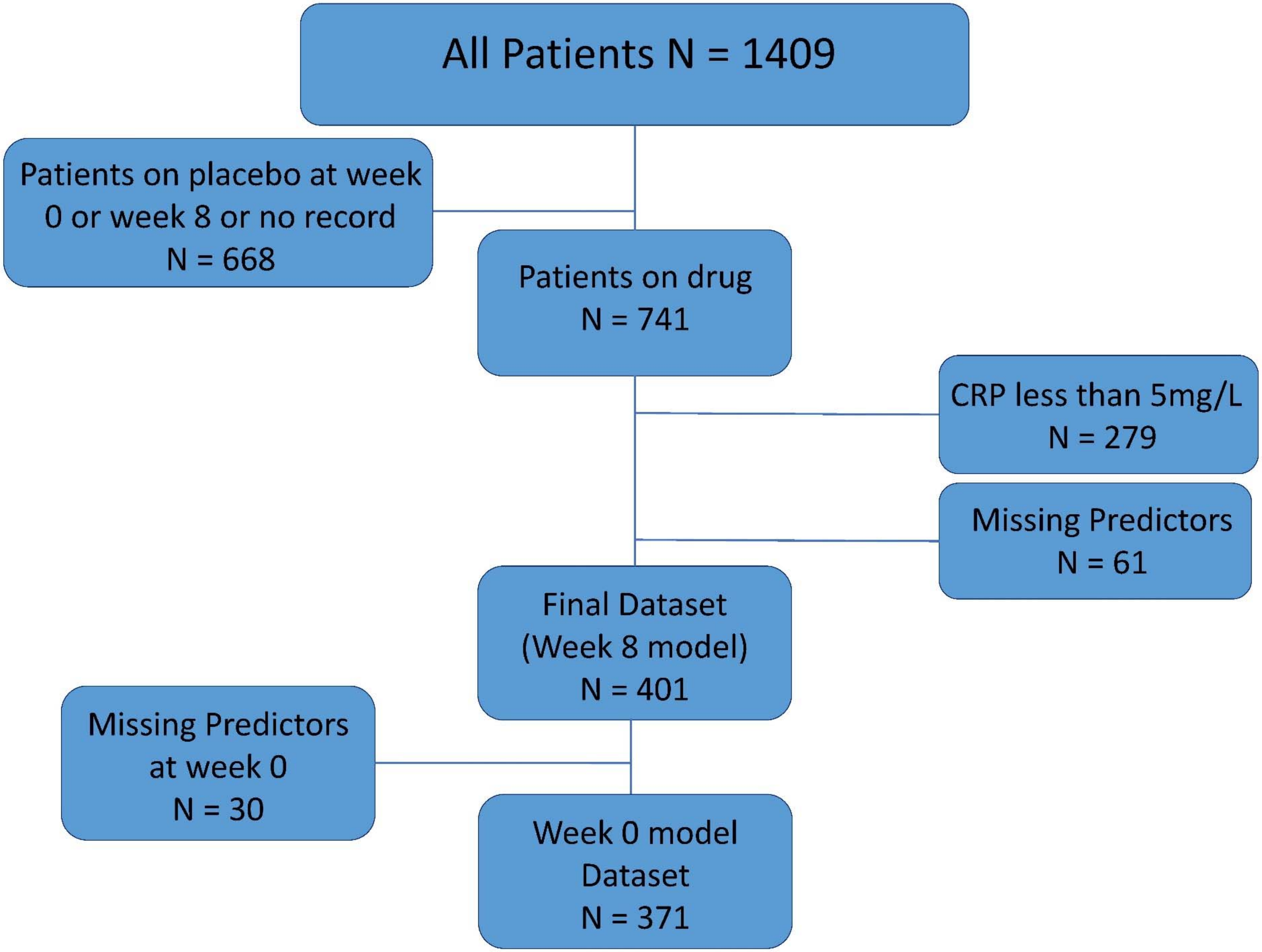

©2019 Waljee AK et al. *JAMA Network Open.*

eFigure 2. Partial Dependence Plots for Baseline Model Predictors

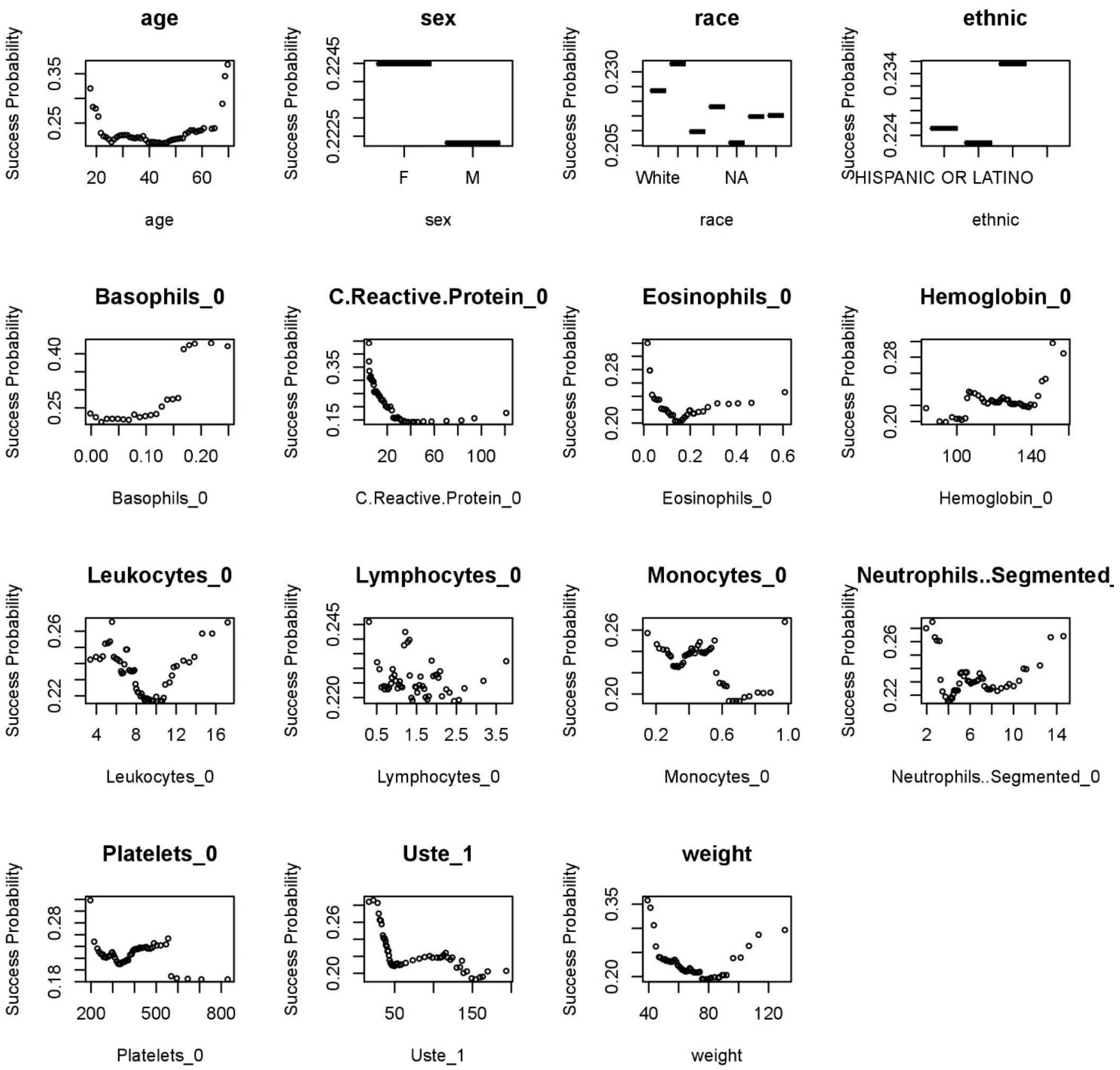

©2019 Waljee AK et al. *JAMA Network Open.*

eFigure 3A. Partial Dependence Plots for Week-8 Model Predictors

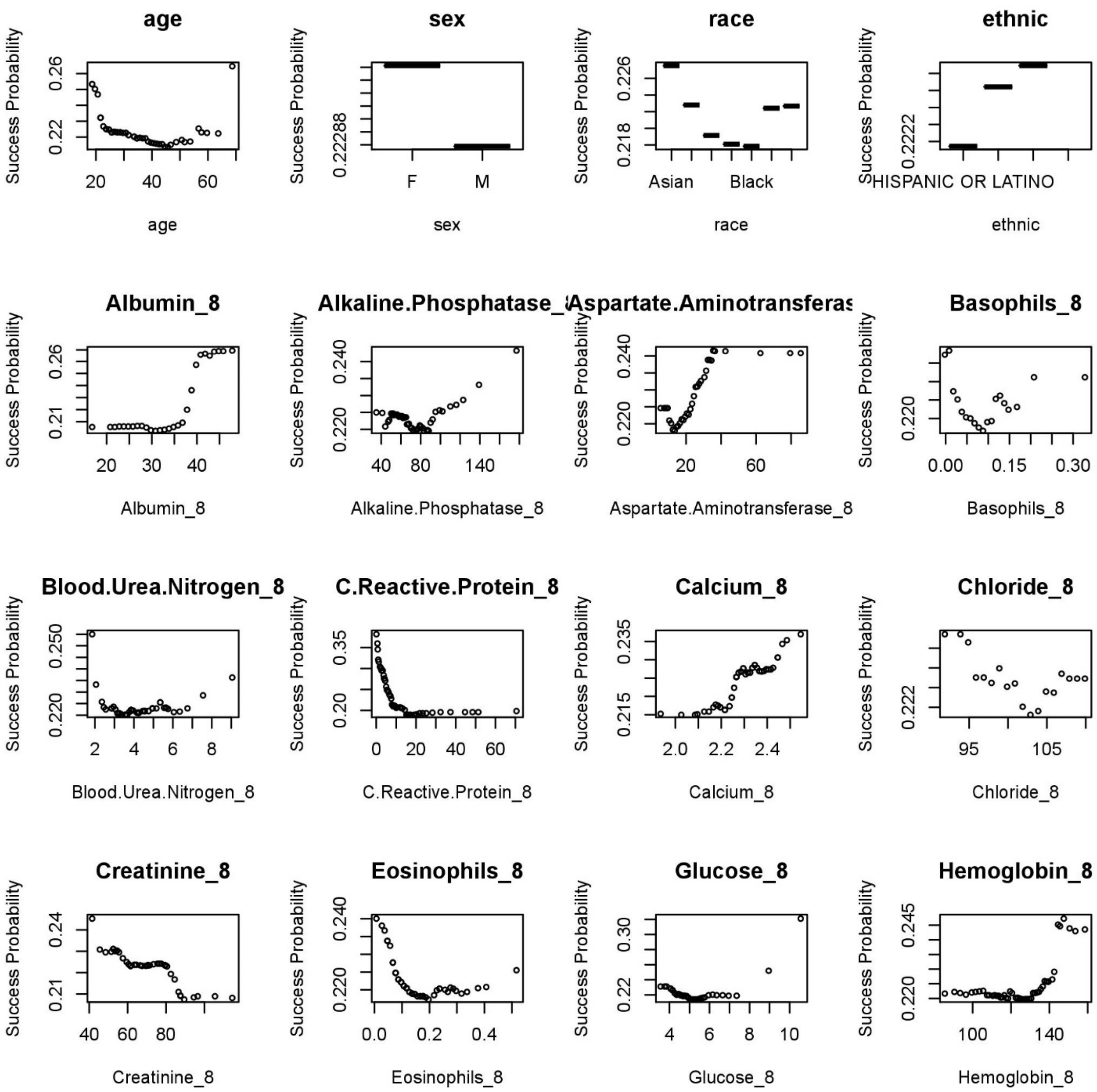

©2019 Waljee AK et al. *JAMA Network Open.*

eFigure 3B. Partial Dependence Plots for Week-8 Model Predictors

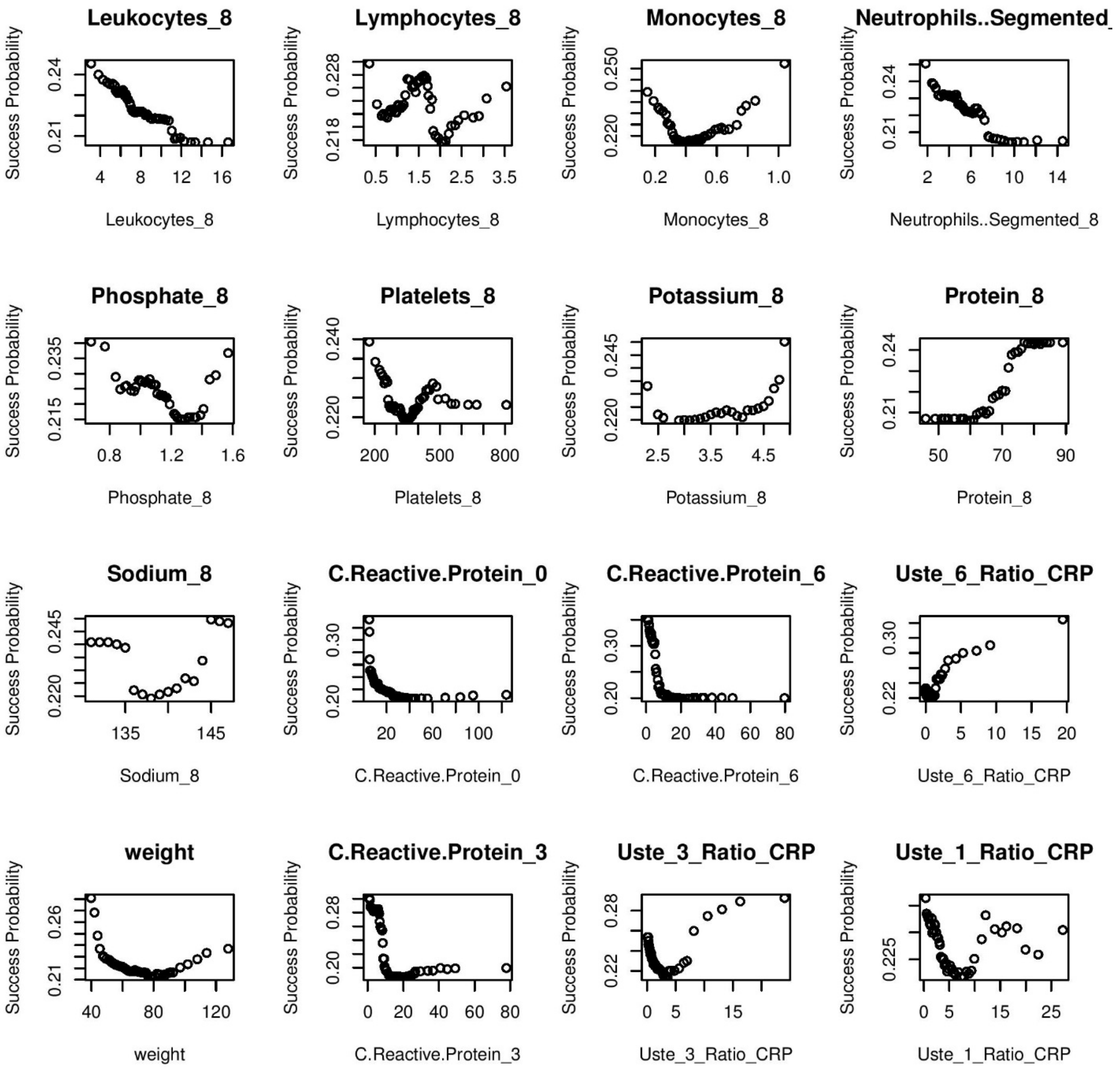

©2019 Waljee AK et al. *JAMA Network Open.*
